# Quantitative Changes in Cerebral Perfusion during Urinary Urgency in Women with Overactive Bladder

**DOI:** 10.1155/2017/2759035

**Published:** 2017-08-17

**Authors:** Nisha G. Arya, Steven J. Weissbart, Sihua Xu, Rupal Bhavsar, Hengyi Rao

**Affiliations:** ^1^Department of Obstetrics and Gynecology, Perelman School of Medicine, The University of Pennsylvania, Philadelphia, PA, USA; ^2^Department of Urology, Stony Brook School of Medicine, Stony Brook, NY, USA; ^3^Center for Functional Neuroimaging, Department of Neurology, Perelman School of Medicine, The University of Pennsylvania, Philadelphia, PA, USA

## Abstract

**Purpose:**

To quantitatively measure changes in cerebral perfusion in select regions of interest in the brain during urinary urgency in women with overactive bladder (OAB) using arterial spin labeling (ASL).

**Methods:**

Twelve women with OAB and 10 controls underwent bladder filling and rated urinary urgency (scale 0–10). ASL fMRI scans were performed (1) in the low urgency state after voiding and (2) high urgency state after drinking oral fluids. Absolute regional cerebral blood flow (rCBF) in select regions of interest was compared between the low and high urgency states.

**Results:**

There were no significant differences in rCBF between the low and high urgency states in the control group. In the OAB group, rCBF (mean ± SE, ml/100 g/min) increased by 10–14% from the low to the high urgency state in the right anterior cingulate cortex (ACC) (44.56 ± 0.59 versus 49.52 ± 1.49, *p* < 0.05), left ACC (49.29 ± 0.85 versus 54.02 ± 1.46, *p* < 0.05), and left insula (50.46 ± 1.72 versus 54.99 ± 1.09, *p* < 0.05). Whole-brain analysis identified additional areas of activation in the right insula, right dorsolateral prefrontal cortex, and pons/midbrain area.

**Conclusions:**

Urinary urgency is associated with quantitative increase in cerebral perfusion in regions of the brain associated with processing emotional response to discomfort.

## 1. Introduction

Urinary urgency, a sudden compelling desire to urinate that is difficult to defer, is the main symptom of overactive bladder (OAB), a functional disorder of bladder storage characterized by the symptoms of urinary urgency, usually associated with frequency and nocturia [1]. OAB affects approximately 15% of US women and prevalence rates are higher in women than men [2]. Common treatment options for OAB, such as behavioral and pharmacologic therapy, have limited efficacy [3]. The development of more efficacious treatment for OAB has been limited by a lack of understanding of the underlying pathogenesis of OAB.

While bladder outlet obstruction caused by prostatic enlargement is a common cause of OAB in men, anatomic abnormalities of the urinary bladder have not been identified in women. Brain imaging studies in a related functional bowel disorder, irritable bowel syndrome, have identified abnormalities in the processing of interoceptive stimuli in the brain [4]. A similar abnormality in processing of afferent signals may exist in women with OAB also. In meta-analyses of brain imaging studies, majority performed using BOLD fMRI, we and other authors have previously reported that urinary urgency is associated with activation of several cortical and subcortical regions that comprise the limbic system, that is, region of the brain that processes emotional response to pain and discomfort, such as anterior cingulate cortex (ACC), insula, and prefrontal cortex (PFC) [5, 6]. However, the magnitude of activation in these regions has not been measured; thus the precise relationship between urinary urgency and the limbic cortex remains unclear.

Neural activity in the brain is tightly coupled with regional cerebral blood flow (rCBF). Arterial spin labeling (ASL) is a fMRI technique that allows quantitative measurement of absolute cerebral perfusion [7]. Unlike PET scans that require the administration of radioactive intravenous contrast to measure cerebral perfusion, ASL uses inverted proton spins of magnetically labeled water in the blood as a tracer. Using continuous ASL, Deutsch et al. reported quantitative increases in absolute rCBF in several regions of the brain involved in the pain matrix in women with interstitial cystitis [8]. However, cerebral perfusion changes associated with urinary urgency in women with OAB have not been quantified.

The primary aim of this study is to determine whether CNS processing of sensory signals from the bladder differs in women with OAB and controls. We induced urinary urgency in women using an oral fill protocol. We quantitatively measured absolute rCBF in the low urgency state immediately after voiding and compared it to absolute rCBF in the high urgency state following intake of oral fluids in women with OAB and controls. Our hypothesis is that urinary urgency is associated with increased rCBF in select regions of interest in the brains of women with OAB.

## 2. Methods

Following IRB approval from the University of Pennsylvania, we recruited 12 women with overactive bladder and 10 controls. Inclusion criteria for the OAB group were age ≥ 18 and diagnosis of OAB confirmed by a response of “most of the time” or “all of time” to the following question, “Do you have a sudden need to rush to the toilet to urinate?” Controls were women age 18 or older who responded “never” to the above question. Exclusion criteria for both groups included claustrophobia, metallic device prohibiting MRI, neurologic disease (e.g., multiple sclerosis, Parkinson's disease, and spinal cord injury), bladder pain syndrome, recurrent urinary tract infections, postvoid residual volume > 50 ml, and pregnant or nursing women. Informed consent was obtained from all women. Urinalysis was performed on all women.

### 2.1. Questionnaires

All subjects completed the following questionnaires prior to undergoing ASL imaging: International Consultation on Incontinence Modular Questionnaire-Female Lower Urinary Tract Symptoms (ICIQ-FLUTS), Hospital Anxiety and Depression Scale, and Visual analog scales (VAS) for pain and urgency. The ICIQ-FLUTS questionnaire is a validated 12-item questionnaire that has been widely used to measure lower urinary tract symptoms in women [9]. In this questionnaire, bother from urinary urgency, the cardinal symptom of OAB, is measured on a scale of 0 to 10 with higher scores indicating greater bother. The Hospital Anxiety and Depression Scale is a well validated instrument for measuring anxiety and depression [10]. The anxiety and the depression score each range from 0 to 21 with higher scores suggesting greater anxiety or depression. We used a 10-point VAS to measure bladder pain and urgency during scanning.

### 2.2. ASL Brain Scans

Each subject participating in the study underwent two arterial spin labeling (ASL) brain scans: (1) in the low urgency state immediately after voiding and (2) in the high urgency state after drinking oral fluids. Low urgency state was defined as urgency rating on <3 on a VAS immediately after voiding. High urgency state was defined as urgency as ≥7 on VAS after drinking oral fluids. For the low urgency state ASL scan, women were asked to void, the postvoid residual urine volume was measured using a portable bladder ultrasound, subjects completed the VAS, and then the ASL brain scan was performed. For the high urgency state ASL scan, women were asked to drink 250–500 ml water until they reported urinary urgency of at least 7 on the VAS, bladder volume was measured using the portable bladder ultrasound, and subjects were escorted into the MRI suite for the ASL scan. To reduce confounding caused by anxiety of entering the MRI scanner, the order of the low and high urgency ASL scans was randomly counterbalanced such that half of the subjects underwent the low urgency ASL scan first while half underwent the high urgency ASL scan first. During each ASL scan, women were asked to rate their level of urgency on a visual analog scale of 1–10 on a screen that was projected in the scanner. Anatomical scout MRI imaging for realignment was performed each time the subject was moved into the scanner.

### 2.3. Imaging Parameters and Preprocessing

Imaging data were acquired on a Siemens 3.0 T Trio whole-body scanner (Siemens AG, Erlangen, Germany) with a 32-channel array coil. ASL images were acquired using a pseudo-continuous arterial spin labeling sequence that we have developed and extensively tested at our institute [11–13]. Twenty slices (5 mm thickness, 1 mm gap) were collected using the following parameters: repetition time (*T*_*R*_) = 4 s, echo time (*T*_*E*_) = 18 ms, labeling time = 1.5 s, delay time = 1 s, flip angle = 90°, field of view = 220 × 220 mm^2^, and matrix = 64 × 64. For each ASL scan, we collected 80 acquisitions over 320 s. During the low urgency condition, a high resolution T1-weighted 3D anatomical image was also acquired using the standard MPRAGE (magnetization prepared rapid acquisition gradient-echo) sequence with the following parameters: repetition time (*T*_*R*_) = 1,620 ms; inversion time = 950 ms; echo time (*T*_*E*_) = 3 ms; flip angle = 15°; 160 contiguous slices of 1.0 mm thickness; FOV = 192 × 256 mm^2^; matrix = 192 × 256. Each anatomic scan lasted 6 minutes.

### 2.4. Functional Imaging Data Analysis

Image analysis was performed using Statistical Parametric Mapping (SPM8) software (http://www.fil.ion.ucl.ac.uk/spm, Wellcome Department of Cognitive Neurology, London) based in Matlab R2012b (MathWorks Inc., Natick, MA, USA), with some modification for perfusion analysis developed and validated at our institution (see https://cfn.upenn.edu/perfusion/software.htm) [14]. Preprocessing steps for each subject included motion correction (realignment to the first image of the time series) and coregistration with their respective high resolution anatomical image. Perfusion-weighted image series were then generated by pairwise subtraction of the label and control images, followed by conversion to absolute CBF images based on a single-compartment continuous arterial spin labeling perfusion model [14]. Thus the resulting CBF data set contained 40 acquisitions with an effective *T*_*R*_ of 8 seconds. One mean CBF map was generated for each subject, which was normalized to Montreal Neurologic Institute (MNI) template available in SPM 8 and smoothed in space with a three-dimensional 8 mm full-width-at-half-maximum Gaussian kernel. Group quantitative CBF images were generated for the high urgency state, low urgency state, and the subtraction of the two conditions.

### 2.5. rCBF Calculation

Motion corrected ASL scans were compartmentalized into groups of control and labeled images. Perfusion-weighted images were obtained by pairwise subtraction of the label and control images. Quantitative CBF maps (mean low urgency state rCBF map and a high urgency state CBF map) were calculated from the perfusion-weighted images using a single-compartment continuous ASL perfusion model, *f* = Δ*MλR*_1*a*_exp⁡(*ωR*_1*a*_)/2*M*_*o*_*α* × [1 − exp⁡(–*τR*_1*a*_)] − 1, where *f* is CBF, Δ*M* is the difference signal between the control and label acquisitions, *R*_1*a*_ is the longitudinal relaxation rate of blood, *τ* is labeling time, *ω* is postlabeling delay time, *α* is labeling efficiency, *λ* is the blood/tissue water partition coefficient, and *M*_*o*_ is approximated by control image intensity [13, 14]. The parameters used in this study were *R*_1*a*_ = 0.67 s^−1^, *α* = 0.85, *λ* = 0.9 gm/ml, *τ* = 1.6 seconds, and *ω* = 800 ms.

### 2.6. Region of Interest (ROI) Selection and Statistical Analysis

We selected 10 ROIs a priori based on the existing literature of overactive bladder and urge incontinence and bladder pain syndrome [5, 6, 8]. These areas included ACC, dorsolateral PFC, thalamus, posterior cingulate cortex (PCC), insula, pons/midbrain region, supplementary motor area, sensorimotor area, hippocampus, and middle cingulate cortex. All the anatomical ROIs were obtained from AAL (Automated Anatomical Labeling) library [15] and WFU (Wake Forest University) Pickatlas. ROI analyses were conducted using Marsbar toolbox from SPM and the quantitative global and regional mean CBF values were extracted for all the subjects. For each ROI in each subject, regional absolute CBF values were calculated using the method described above. We compared absolute regional CBF in the low urgency state and high urgency state within each group using paired *t*-test. Demographic data, questionnaire scores, and change in CBF between groups were compared using nonparametric *t*-test. SPSS Statistics for Windows, Version 24.0 (Armonk, NY, IBM Corp.), was used for analysis.

In addition, to explore whole-brain analysis, we used general linear modeling in SPM to evaluate rCBF pattern changes from the low urgency state to the high urgency state. This analysis checked for areas of activation in the entire brain not just defined ROIs or specific hypothesis. Paired *t*-test statistical map was generated comparing mean CBF images from low urgency to high urgency state. Activation clusters were identified at a significance level of uncorrected *p* < 0.005 with a cluster extent threshold of 40 voxels. Significant clusters were reported if they met family wise error (FWE) correction at peak level of *p* < 0.05 to correct for multiple comparisons.

## 3. Results

The demographic data for women with OAB and controls is presented in Table 1. There was no significant difference in the age of women with OAB and controls. Controls had significantly lower BMI than women with OAB. The mean urgency bother score was significantly higher for women with OAB than controls. There was no significant difference in anxiety and depression scores and the postvoid residual volume of women with OAB and controls. Urinalysis was negative for all women. The mean bladder volume during the high urgency state was significantly lower in women with OAB than controls.

No subject reported anxiety during MRI scanning, and all women completed both ASL scans.

**Figure 1:**
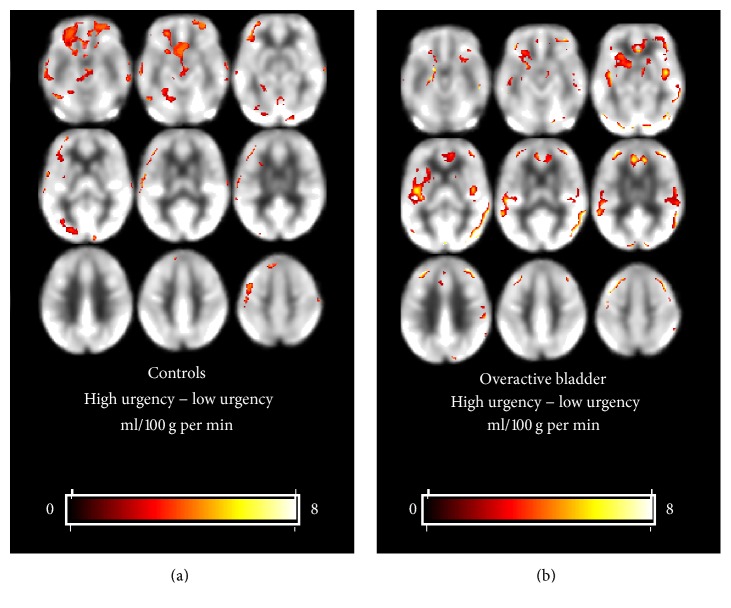
Subtraction image of rCBF differences between high urgency state and low urgency state for control and overactive bladder groups. No significant differences in rCBF are noted between the high and low urgency states for control subjects. The overactive group shows significant increase in rCBF (in yellow) in the right ACC and left ACC and the left insula ((b) second row). The *xyz* coordinates of peak activation were as follows: right ACC (12, 40, 6), left ACC (−8, 20, and 16), and left insula (−28, 20, and −10).

Figure 1 shows subtraction images of rCBF differences between high urgency state and low urgency state for control and OAB groups, respectively, from the ROI analysis. No significant differences in rCBF between high urgency state and low urgency state were noted for control subjects. The OAB group showed significant increase in rCBF from the low urgency state to the high urgency state in the right and left ACC and the left insula.

Table 2 shows absolute rCBF data (ml/100 g of brain tissue/min) during the high and low urgency states in select region of interests that had been identified a priori for the OAB and control groups. No significant differences in absolute rCBF between the high and low urgency states were noted in the control group. In the OAB group, absolute rCBF in the right ACC, left ACC, and the left insula was significantly higher in the high urgency state than the low urgency state. The change in rCBF from the low to the high urgency state in the right ACC, right dorsolateral prefrontal cortex (DL PFC), and left thalamus was significantly greater in the OAB group than the control group.

**Figure 2:**
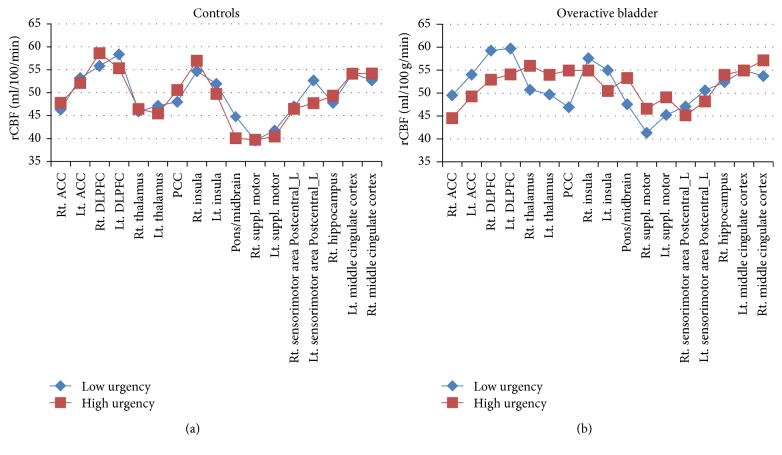
Absolute rCBF for several regions of interest in the high and low urgency states for the overactive bladder and control groups. rCBF does not vary much between the high and low urgency states for controls but was 10–14% higher in the right ACC, left ACC, and left insula for the OAB group.

The absolute CBF data in Table 2 are represented graphically in Figure 2. In the control group, absolute rBCF did not vary much between the low and high urgency states. In the OAB group, absolute rCBF was 10–14% higher in the right ACC and left ACC and the left insula in the high urgency state as compared to the low urgency state.

**Figure 3:**
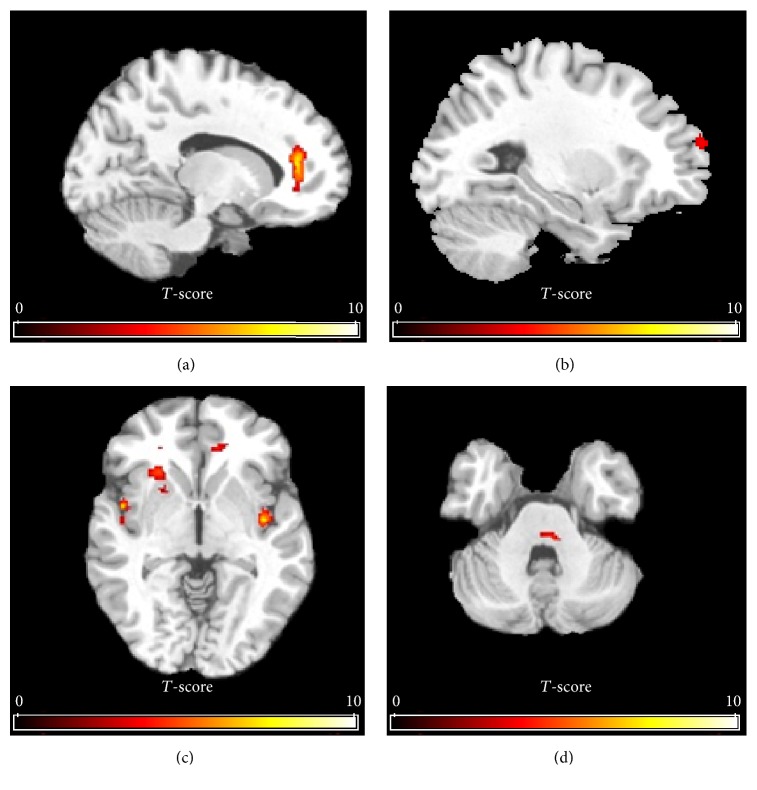
Group activation maps of mean cerebral blood flow (CBF) changes between empty and full bladder state using SPM in subjects with overactive bladder. Note the increase in CBF (in yellow) in anterior cingulate cortex (a), right dorsolateral prefrontal cortex (b), left and right insula (c), and pons/midbrain (d). Note the substantial overlap with regions showing increased rCBF from the region of interest analysis in Figure 1 (ACC and insula).

Figure 3 shows the group activation map of changes in CBF using a non-hypothesis-driven approach in SPM for the OAB group. Many of the regions identified through this whole-brain analysis overlapped with the findings of the ROI analyses (right and left ACC and left insula). Additional areas of activation were identified in the right insula, right dorsolateral (PFC), and pons/midbrain area. No deactivation clusters were found at uncorrected *p* < 0.005.

## 4. Discussion

The main advantage of ASL fMRI is that it allows quantitative measurement of cerebral perfusion using a noninvasive technique and does not require repeated filling and emptying the bladder [7, 8]. To our knowledge, this is the first study using ASL to investigate cerebral perfusion changes in women with overactive bladder. Using ASL, we found that, in women with OAB, urinary urgency is associated with 10–14% increase in absolute regional CBF in key regions of the brain that comprise the limbic system: the ACC and the insula. Secondary non-hypothesis-driven approach using SPM substantiated our findings and identified additional areas of activation in the dorsolateral PFC and the pons/midbrain area. Despite greater bladder filling volumes during the high urgency scan, control subjects did not show increased perfusion in any of our prespecified regions of interest. Our findings suggest that, in women with OAB, urinary urgency is associated with abnormal processing of sensory input in the limbic cortex.

Our findings advance understanding of the pathogenesis of urinary urgency. Using continuous ASL, Deutsch et al. reported that, in women with interstitial cystitis, bladder filling is associated with activation of several regions of the brain involved in the pain matrix such as the supplementary motor area, motor and sensory cortex, insula, hippocampus, and middle and posterior cingulate areas [8]. We found that, in women with OAB, urinary urgency is associated with significant increase in cerebral perfusion in key regions of the limbic system, that is, the ACC and insula. The insula, an area of the cerebral cortex that lies deep into the lateral sulcus, receives afferent homeostatic information of “interoception” from internal visceral processes, such as heart rate, digestion, and micturition, and is also called the sensory cortex of the autonomic nervous system [16]. The ACC on the medial surface of the frontal lobe is also called the motor cortex of the autonomic nervous system because it is responsible for emotional, behavioral, and motor responses to visceral stimuli [17]. In women with OAB, ACC activation is associated with the motor response of pelvic floor contraction through recruitment of accessory pathways [6]. Most fMRI studies have reported activation of both the insula and the ACC during urinary urgency in women with OAB though Komesu et al. reported activation of the ACC only [18]. In our study, the magnitude of change in absolute rCBF (4.5 to 4.96 ml/100 g/min) in the OAB group was similar in the ACC and the insula. Our findings suggest that activation of both the insula and the ACC is a key component of urinary urgency experienced by women with OAB and likely represents both afferent sensation of bladder filling and an emotional and motor response to the threatened loss of urine.

We identified additional areas of activation in the dorsolateral PFC and the pons/midbrain region in women with OAB on secondary non-hypothesis-driven analysis (Table 1). Of these, the PFC is particularly important because change in absolute rCBF in the PFC was significantly greater in OAB subjects than controls on region of interest analysis. The PFC participates in cognitive activities and is involved with monitoring conflict and decision making in an emotional context, that is, urgency in inappropriate social situations [6]. These findings suggest that urgency may represent an emotional response to discomfort and potentially explains the success of techniques such as distraction in preventing leakage. The activation in the pons/midbrain likely represents the periaqueductal gray (PAG) where sensory information from the bladder is integrated [6]. Overall, our findings of quantitative increase in rCBF in the limbic cortex (ACC, insula), PFC, and brainstem substantiate existing conceptual models of sensory processing in the brain during urgency [19].

Our findings in control subjects differ from some existing fMRI studies. Prior fMRI studies have identified activation of the insula in continent controls [20]. In our study, though cerebral perfusion was higher in the insula during the high urgency state than the low urgency state in controls, this difference did not reach significant levels. Our findings may have been due to high variability of observed measurements and relatively small sample size. Alternatively, our lack of observed differences in rCBF measurements in controls subjects may be related to differences in populations and protocols of bladder filling. Unlike prior fMRI studies that have measured brain activation in mostly young women with OAB at maximum cystometric capacity using a urinary catheter, we measured brain activation during urinary urgency in older controls. Activation of the insula may be more prominent in younger subjects [21]. Also, the volume of filling that induces urgency may not correspond to bladder capacity. Similar to our study, Komesu et al. also measured brain activation during urgency rather than at cystometric capacity. In that study also, activation of ACC and insula was not prominent in continent controls at higher levels of urgency. The advantage of measuring brain activation during urinary urgency rather than with cystometric capacity is that it may help to elucidate mechanisms of a functional syndrome that is defined based on symptoms such as urinary urgency rather than objective findings such as cystometric capacity.

Our finding that changes in cerebral perfusion can be quantitatively measured in women with OAB has potentially useful clinical applications. Deutsch et al. have proposed that quantitative measurement of cerebral perfusion could be used for the diagnosis of complex cases of bladder pain syndrome [8]. A similar diagnostic use is applicable for complex patients of OAB. While the vast majority of cases of OAB can be diagnosed based on clinical symptoms alone, women with overlapping symptoms of OAB and bladder pain pose both a diagnostic and therapeutic challenge [22]. The current “gold standard” for the diagnosis of OAB, detrusor overactivity during filling cystometry, has a sensitivity of only 64% and a specificity of 67% [23]. A quantitative biomarker that is linked to the underlying pathogenesis of the condition would allow a more specific diagnosis in complex cases. Objective measurement of cerebral perfusion could also be used to develop novel treatments for OAB such as hypnosis and transcranial magnetic stimulation that target cerebral perfusion in specific regions of interest and have been used in the treatment of depression [24].

Strengths of our study include careful inclusion of well characterized cohort of women with OAB with absence of confounding from factors such as anxiety that could cause activation of the limbic cortex. Unlike BOLD fMRI, ASL does not require repeated emptying and filling of the bladder. This advantage of ASL allowed us to use an oral fill protocol similar to that used by Deutsch et al. [8]. The advantage of an oral fill protocol is that it prevents false positive afferent signals induced by placement of a catheter, an issue that could be particularly important in conditions such as OAB that are characterized by increased afferent signaling. The disadvantage of this protocol is that it does not allow bladder filling volumes to be precisely controlled. The type of fill protocol used during imaging studies will depend on the goals of the project; catheter based protocols may be better for studies that require precise measurement of bladder volumes while oral protocols may be better for studies focusing on reproducing patient symptoms such as pain or urgency.

In recent years, several neuroimaging studies, most using functional MRI, have advanced our understanding of how afferent signals from the bladder are processed in the brain. Of these, BOLD fMRI and ASL have gained wide applications because both use endogenous tracers and are therefore noninvasive. The advantage of ASL over BOLD fMRI is that it allows quantitative measurement of changes in CBF and has better spatial localization with the actual site of the brain activated by a specific task [25]. The limitation of ASL as compared to BOLD has a lower signal noise ratio and is thus less sensitive to changes in CBF induced by a given task [25, 26]. BOLD fMRI also has higher temporal resolution than ASL, making it more suited to event-related designs, for example, in a language task, and is technically easier to implement since it does not usually require any additional programming of the radio frequency and gradient pulses provided by the manufacturer. Combined BOLD and ASL techniques may help to achieve high spatiotemporal resolution [27].

Our study using ASL makes a significant contribution by demonstrating quantitative changes in rCBF in specific regions of the brain associated with processing emotional response to discomfort.

## 5. Conclusions

Functional neuroimaging using arterial spin labeling can noninvasively quantify perfusion changes in the brain using a noncatheterized oral fluid challenge paradigm. In women with OAB, urinary urgency is associated with increased perfusion in the limbic system known to process emotional response to discomfort.

## Figures and Tables

**Table 1:** Demographic data of subjects with overactive bladder and controls.

	Overactive bladder (*n* = 12) (median ± IQR)	Controls (*n* = 10) (median ± IQR)	*p* value^†^
Age (years)	52.5 ± 16	48 ± 18	0.42
BMI (kg/m^2^)	35.1 ± 11.5	32 ± 12	<0.05
Urgency bother score (0–10)^1^	8.5 ± 3	0 ± 1	<0.001
Bladder pain score (0–10)^2^	0 ± 2	0 ± 1	0.76
Anxiety score^3^	3.5 ± 9	4.0 ± 9	0.41
Depression score^3^	4.5 ± 8	4.5 ± 7	0.32
Postvoid residual volume (ml)	45 (±55)	30 (±25)	0.66
Bladder volume during high urgency scan (ml)	455 (±350)	850 (±250)	<0.01

^†^Nonparametric *t*-test; ^1^International Consultation on Incontinence Modular Questionnaire-Female Lower Urinary Tract Symptoms (ICIQ-FLUTS); ^2^by visual analog scale; ^3^by Hospital Anxiety and Depression Scale.

**Table 2:** Absolute regional CBF in the low and high urgency state in select ROIs in women with overactive bladder and controls (ml/100 g/minute).

Region of interest (ROI)	Controls			Overactive bladder		
	Low urgency	High urgency	Δ (High − low urgency)	Low urgency	High urgency	Δ (high − low urgency)
Rt. ACC	47.79 ± 1.46	46.34 ± 1.20	−1.45 ± 1.61	44.56 ± 0.59	49.52 ± 1.49	4.96 ± 1.79^**∗**†^
Lt. ACC	52.12 ± 1.45	53.19 ± 1.48	1.07 ± 2.20	49.29 ± 0.85	54.02 ± 1.46	4.73 ± 1.69^**∗**^
Rt. DL PFC	58.60 ± 1.29	55.81 ± 1.39	−2.79 ± 1.88	52.92 ± 2.08	59.28 ± 1.81	6.35 ± 3.22^†^
Lt. DL PFC	55.31 ± 1.85	58.35 ± 2.29	3.03 ± 3.34	54.09 ± 2.23	59.74 ± 1.63	5.65 ± 2.79
Rt. thalamus	46.46 ± 1.51	45.93 ± 2.73	−0.53 ± 2.99	55.96 ± 2.58	50.70 ± 2.04	−5.25 ± 2.67
Lt. thalamus	45.48 ± 1.46	47.15 ± 1.71	1.69 ± 1.96	53.99 ± 2.08	49.68 ± 1.74	−4.31 ± 2.28^†^
PCC	50.59 ± 2.61	48.08 ± 1.50	−2.56 ± 3.38	55.00 ± 2.10	46.90 ± 2.90	−8.06 ± 4.58
R. insula	54.70 ± 2.07	56.98 ± 2.4	2.28 ± 3.61	54.94 ± 1.67	57.59 ± 1.30	2.64 ± 1.74
L. insula	49.72 ± 1.67	51.93 ± 1.04	2.21 ± 3.51	50.46 ± 1.72	54.99 ± 1.09	4.53 ± 1.64^**∗**^
Pons/midbrain	40.07 ± 2.83	44.76 ± 1.63	4.69 ± 3.60	53.32 ± 2.86	47.56 ± 3.14	−5.75 ± 5.17
R. SMA	39.72 ± 1.04	39.63 ± 1.89	−0.09 ± 1.83	46.61 ± 2.84	41.35 ± 4.19	−5.26 ± 3.69
L. SMA	40.42 ± 0.90	41.76 ± 2.52	1.34 ± 2.48	49.09 ± 3.03	45.25 ± 3.45	−3.85 ± 2.00
R. sensorimotor area	46.47 ± 1.08	46.99 ± 1.80	0.51 ± 2.18	45.14 ± 2.26	47.06 ± 2.35	1.92 ± 3.40
L. sensorimotor area	47.70 ± 1.86	52.68 ± 2.10	4.98 ± 3.77	48.17 ± 2.07	50.61 ± 2.01	2.44 ± 1.91
R. hippocampus	49.36 ± 1.76	47.81 ± 2.60	−1.55 ± 2.33	53.99 ± 3.30	52.41 ± 2.15	−1.58 ± 4.15
R. middle cingulate cortex	54.13 ± 1.63	54.22 ± 1.34	0.09 ± 1.63	54.95 ± 2.11	53.73 ± 2.12	−1.22 ± 3.55
L. middle cingulate cortex	54.19 ± 1.30	52.72 ± 1.55	−1.47 ± 1.29	57.13 ± 2.77	54.93 ± 1.83	−2.20 ± 4.20

^*∗*^ *p* < 0.05 (paired *t*-test comparing rCBF in high to low urgency state within each group); ^†^*p* < 0.05 (nonparametric *t*-test comparing change in rCBF in overactive bladder and control groups); ACC: anterior cingulate cortex, DL PFC: dorsolateral prefrontal cortex; SMA: supplementary motor area; R: right, L: left.

## Abbreviations

ACC: Anterior cingulate cortex
ASL: Arterial spin labeling
CBF: Cerebral blood flow
OAB: Overactive bladder
PFC: Prefrontal cortex
PCC: Posterior cingulate cortex
pCASL: Pseudo-continuous arterial spin labeling.
